# Approach-avoidance activation without anterior asymmetry

**DOI:** 10.3389/fpsyg.2014.00192

**Published:** 2014-03-11

**Authors:** Andero Uusberg, Helen Uibo, Riti Tiimus, Helena Sarapuu, Kairi Kreegipuu, Jüri Allik

**Affiliations:** ^1^Department of Experimental Psychology, Institute of Psychology, University of TartuTartu, Estonia; ^2^Estonian Academy of SciencesTallinn, Estonia

**Keywords:** anterior alpha asymmetry, late positive potential, affective images, motivation, individual differences

## Abstract

Occasionally, the expected effects of approach-avoidance motivation on anterior EEG alpha asymmetry fail to emerge, particularly in studies using affective picture stimuli. These null findings have been explained by insufficient motivational intensity of, and/or overshadowing interindividual variability within the responses to emotional pictures. These explanations were systematically tested using data from 70 students watching 5 types of affective pictures ranging from very pleasant to unpleasant. The stimulus categories reliably modulated self-reports as well as the amplitude of late positive potential, an ERP component reflecting orienting toward motivationally significant stimuli. The stimuli did not, however, induce expected asymmetry effects either for the sample or individual participants. Even while systematic stimulus-dependent individual differences emerged in self-reports as well as LPP amplitudes, the asymmetry variability was dominated by stimulus-independent interindividual variability. Taken together with previous findings, these results suggest that under some circumstances anterior asymmetry may not be an inevitable consequence of core affect. Instead, state asymmetry shifts may be overpowered by stable trait asymmetry differences and/or stimulus-independent yet situation-dependent interindividual variability, possibly caused by processes such as emotion regulation or anxious apprehension.

## Introduction

According to the popular biphasic motivational account, anterior alpha power asymmetry reflects prefrontal lateralization of approach-avoidance processes (for reviews see Coan and Allen, 2004; Harmon-Jones et al., 2010; Miller et al., 2013). However, this model is challenged by occasional failures to find expected asymmetries in response to affective images (see Harmon-Jones et al., 2010). Such null findings are at odds with the general validity and reliability of picture stimuli for eliciting core affect, a construct closely related to biphasic motivation (Bradley and Lang, 2007). This conundrum has been related to (a) limited motivational intensity of, and/or (b) individually variable reactions to affective images (Harmon-Jones et al., 2006; Gable and Harmon-Jones, 2008). The present study systematically tests these explanations using (a) the late positive potential (LPP) together with affective ratings to measure motivational intensity; and (b) a mixed model statistical analysis to detect individual differences.

Anterior asymmetry is usually expressed as the difference between log-transformed spectral energy within the alpha band (8–13 Hz) of the electroencephalographic (EEG) signal recorded from right and left frontal scalp locations (for methodological reviews see Allen et al., 2004; Hagemann, 2004). The actual neural origins of alpha asymmetry remain somewhat obscure (Miller et al., 2013). An initial suggestion that alpha asymmetry reflects a clear lateralization of approach and avoidance systems in anterior brain areas has not been confirmed by modern neuroimaging studies (Murphy et al., 2003; Wager et al., 2003). More nuanced proposals associate anterior alpha as well as components of biphasic motivation with specific regions of the lateral prefrontal cortex (Davidson, 2004; Engels et al., 2007; Shackman et al., 2009; Herrington et al., 2010). However, these accounts remain to be integrated with modern theories interpreting alpha in terms of active inhibition rather than passive idling (e.g., Klimesch et al., 2007; Palva and Palva, 2007; cf. Parvaz et al., 2012; Miller et al., 2013).

While the neuroanatomical sources of asymmetry remain difficult to pinpoint, conceptual correlates of this measure have been gradually deduced. Research of both intra- as well as interindividual asymmetry variance (i.e., state and trait asymmetry; Coan and Allen, 2002, 2004) converges on the idea that positive scores (reflecting more alpha over right than left hemisphere) indicate relatively higher approach-related activity whereas negative scores correspond to the dominance of avoidance processes (Allen and Kline, 2004; Cacioppo, 2004; Coan and Allen, 2004; Harmon-Jones et al., 2010). For instance, positive trait asymmetry correlates with the pleasantness of subsequent affective experiences (e.g., Tomarken et al., 1990; Sutton and Davidson, 2000) as well as reduced risk of affective disorders (Thibodeau et al., 2006). Meanwhile, laboratory manipulations of approach and avoidance motivation can induce positive and negative shifts in concurrent asymmetry, respectively (e.g., Davidson and Fox, 1982; Sobotka et al., 1992; Miller and Tomarken, 2001; Schmidt and Trainor, 2001; Papousek and Schulter, 2002). However, as will be explained next, some data do not align with this biphasic account of asymmetry.

Published (and possibly several unpublished) records exist of failures to find expected state asymmetry effects from EEG responses to affective images (Elgavish et al., 2003; Harmon-Jones et al., 2006; Gable and Harmon-Jones, 2008; Huster et al., 2009; Gable and Poole, 2012). These null findings pose a conceptual challenge to the dominant asymmetry framework. Affective images constitute a reliable method for inducing core affect (Bradley and Lang, 2007)—a subjectively felt bodily state of pleasure and displeasure with some degree of arousal (Posner et al., 2005; Barrett and Bliss-Moreau, 2009). On a conceptual as well as a neurophysiological level, core affect and biphasic motivation are deeply interwoven (Watson et al., 1999; Posner et al., 2005; Bradley and Lang, 2007; Barrett and Bliss-Moreau, 2009; Lang, 2010; Norris et al., 2010; Hamann, 2012). Both phenomena are needed to provide an organism with direction and force for appropriate action in face of threats and rewards. Consequently, stimuli that generate core affect should in most cases inevitably induce some degree of approach or avoidance motivation. According to the biphasic motivational asymmetry model, these stimuli should therefore also lead to changes in state asymmetry (Davidson et al., 1990; Carver et al., 2000). It is thus important to understand why affective images that are widely used to induce core affect occasionally fail to shift anterior asymmetry.

Several explanations are possible. One possibility with important implications for asymmetry theories is that these failures constitute a rare example of successfully induced core affect as well as biphasic motivation remaining invisible in EEG asymmetry. Before considering this intriguing possibility, however, a number of alternative explanations should be ruled out. First of all, the null findings may simply originate from insufficient signal to noise ratio or statistical power. Expected asymmetries have indeed been induced by pictures after assuring sufficient reliability, although at distributed rather than selectively anterior scalp locations (Huster et al., 2009). Among more specific possibilities, affective images may sometimes fall short of activating core affect/biphasic motivation, inducing merely cognitive processes such as stimulus categorization (e.g., Harmon-Jones et al., 2006). Alternatively, the absence of an expected effect at the sample level may be an illusion caused by stimulus-dependent individual differences. For instance, positive asymmetries from some participants may cancel out negative ones measured from others on a grand average level analyzed in most studies. Both specific mechanisms were illustrated in a study where pictures of desserts generated more positive asymmetries than neutral images, but only for hungrier and more dessert-liking subjects (Gable and Harmon-Jones, 2008). In this experiment, revealing the expected effects thus required (a) amplification of the motivational relevance of the pictures by state hunger or trait-like dessert-preference, and (b) separation of participant groups experiencing different levels of motivational intensity.

Previously, the roles of motivational intensity and individual differences in picture-induced asymmetry have mostly been studied using specific emotional states such as hunger (Gable and Harmon-Jones, 2008; Harmon-Jones et al., 2011) or anger (Harmon-Jones et al., 2006; Gable and Poole, 2012). However, these very specific states involve several component processes beyond core affect and biphasic motivation. The generalizability of the proposed explanations for the affective images conundrum therefore remains to be established. To this end, the present study investigates responses to semantically more or less heterogeneous picture sets constructed to induce homogenous forms of core affect/biphasic motivation. We re-analyzed EEG from an experiment where participants viewed affective images from five categories while making affective evaluations (Uusberg et al., 2013). The data were collected from a large sample (*n* = 70) and are sufficiently lengthy (around 50 s per average) thereby assuring sufficient statistical power and signal to noise ratio, respectively (cf. Huster et al., 2009).

To test the possibility that emotional pictures fail to induce asymmetry due to their limited motivational relevance (Harmon-Jones et al., 2006), we analyzed subjective affective ratings as well as LPP amplitudes. Self-reported valence and arousal ratings can be considered psychometric proxies of biphasic motivational intensity (Bradley and Lang, 2007; Lang, 2010). The LPP meanwhile is a positive voltage deflection over central-posterior scalp areas that is amplified by emotionally arousing pleasant as well as unpleasant stimuli while remaining fairly independent of low-level perceptual features as well as stimulus repetitions (e.g., Codispoti et al., 2007; for reviews see Schupp et al., 2006; Olofsson et al., 2008; Hajcak et al., 2010). It is thought to reflect enhanced cortical processing of motivationally significant stimulus representations (Sabatinelli et al., 2007), triggered by both cortical as well as subcortical bias signals (Sabatinelli et al., 2013). The LPP is thus well suited for capturing the intensity of the motivational state whose direction is reflected in asymmetry (Gable and Poole, 2012; Gable and Harmon-Jones, 2013). Therefore, in case affective pictures indeed fail to induce asymmetry due to their limited motivational relevance, they should also leave the LPP amplitude unamplified.

We also explicitly analyzed the possibility that the absence of sample level asymmetry effect conceals individually different responses to heterogeneous picture stimuli (Gable and Harmon-Jones, 2008). Instead of confining these analyses to any preconceived trait dimension (e.g., gender or Extraversion) that may or may not be relevant for asymmetry, we quantified all individual differences present in the data. More specifically, the experimentally measured variance was decomposed into four additive components (Stemmler and Wacker, 2010):

(1) Y=S+I+SI+ε

In this model, beyond the residual variance ε an outcome variable *Y* (e.g., asymmetry) is defined by the main effect of stimulus variability *S*, the main effect of interindividual variability *I* and the interaction between the stimuli and the individuals *SI*. This decomposition is particularly useful for current purposes as the *SI* component isolates dependent variable shifts occurring in response to some stimuli only in some participants—exactly the pattern suggested to conceal affective main effects in asymmetry studies (Gable and Harmon-Jones, 2008). The remaining components meanwhile capture stimulus-independent differences between participants (*I*) as well as the affective main effect itself (*S*).

## Materials and methods

### Participants and procedure

The analyzed sample consisted of 70 healthy, right-handed university students and recent graduates (age *M* = 20.7, *SD* = 2.1, range 18–29 years, 28 men). Seven participants were excluded from analyses involving F8-F7 asymmetry due to low segment retention rate after artifact correction (<50% of trials, see below for details). Informed consent was assured before arrival at the lab. The experiment took place in a silent and dimly lit room. Participants were comfortably seated at 114 cm from a 14-inch computer screen. After electrode placement, instructions, and practice trials the participants remained alone in the room.

### Stimuli and design

Images from the International Affective Picture System (IAPS; Lang et al., 2005) were used to construct five affectively homogenous categories: neutral (e.g., household objects, landscapes); pleasant (e.g., children, desserts); unpleasant (e.g., weapons, snakes); high arousal (HA) pleasant (erotic opposite sex couples to maximize relevance for both hetero- and homosexual males and females); high arousal (HA) unpleasant (mutilated human bodies; for a list of the images see Uusberg et al., 2013). Mean normative valence ratings of image categories increased from the HA unpleasant to HA pleasant category while arousal ratings formed three distinct levels—HA pleasant and unpleasant pictures; low arousal pleasant and unpleasant ones and neutral baseline images. These image selection criteria were largely upheld in the ratings given by the current sample (see Results). The images were presented in two conditions. In the affective evaluation condition participants rated the valence and arousal of the affective states generated by each image. In the nonaffective condition evaluations of stimulus luminosity and object numerocity were required. Only data from the affective task are analyzed in this paper as the nonaffective task was designed to reduce the intensity of affective experiences. Prior to the affective task, participants were trained to use the valence scale to express the “negativity-positivity or pleasantness-unpleasantness of the emotional state experienced during picture viewing.” During the experiment, the scale was presented with the title “valence” and end-point labels “neg” and “pos.” The arousal instruction asked the participants to rate the “strength-weakness of the emotional state experienced during picture viewing.” The scale was presented with the title “intensity” and labels “min” and “max.”

Two sets of 60 images (12 from each category) were constructed with similar affective ratings, semantic content, and picture orientation to be presented in the two conditions. The order of conditions as well as condition and image set pairing were counterbalanced between participants. All pictures from one set were presented in pseudo-randomized order with one task instruction in one experimental block. Each block was repeated 3 times before switching to the other condition. The stimuli were presented on a computer screen with angular size of 15.24° horizontally and 11.52° vertically. A single trial started with a fixation cross presented for 1500 ms in the middle of a dark gray screen followed by the stimulus for 1500 ms. Images from one affective category were thus presented for 54 s in total (3 repetitions of 12 images for 1.5 s each). Upon stimulus offset, two 9-point response scales were presented consecutively for unlimited time. Participants responded using a computer keyboard. Further details of the methods are available in a previous publication (Uusberg et al., 2013).

### EEG recording and processing

Continuous EEG was recorded from 30 scalp, 4 ocular, and two earlobe reference electrodes. Offline processing was implemented using EEGLAB (Delorme and Makeig, 2004) software. After re-sampling to 256 Hz the data were low-pass filtered at 45 Hz to remove electrical line noise. Independent Component Analysis (ICA) was used to remove eye-movement artifacts. An Informax ICA solution was obtained from high-pass filtered (1 Hz; 12 dB/oct) training copy of the data cleaned of gross artifacts via channel (EEGLAB rejchan; probability >5*SD*) as well as epoch rejection algorithms (rejspec; 20–40 Hz; < −100 and >25 dB). Components containing known features of eye-blinks as well as horizontal and vertical eye movements were rejected for each participant (*M* = 3.6, *SD* = 0.87, range 2–6) before reconstructing the continuous unfiltered data (Debener et al., 2010). The ICA-pruned data were cut into 3000 ms segments covering 1500 ms before and after stimulus onset with −200 ms removed as baseline. Remaining artifacts were removed using spectral (15–30 Hz, < −30 or >30 dB) and threshold (±125 μV) criteria applied only to the channels analyzed in this paper (F3, F4, F7, F8, CP1, CP2, P3, P4, PO3, PO4, and Pz). On average 94.6% of the data (range 69.4–100%, *SD* = 6.3) or 51.1 s per affective category were retained. The retention rate did not depend on stimulus category [*F*_(4, 276)_ = 1.00, *p* = 0.41].

Spectral power estimates were calculated from concatenated post-stimulus sections of epochs for each stimulus type using Fast Fourier Transforms (FFT, 1 s Hamming windows; 50% overlap; zero-padding for 0.5 Hz resolution). As specific boundaries of alpha oscillations may vary between individuals (Klimesch, 1999) the analyzed frequency range was defined individually for each participant as 0.8–1.3^*^IAF, where IAF is the visually determined individual alpha peak frequency (range 7.5–11.5, *M* = 9.98, *SD* = 0.84; Doppelmayr et al., 1998). Data averaged in fixed frequency windows yielded similar results which are therefore not reported in this paper. To obtain asymmetry estimates, power values averaged within individually defined boundaries from the mid-frontal F4 and F3 and lateral-frontal F8 and F7 electrodes were natural log transformed and the left estimate subtracted from the right one (Allen et al., 2004). These locations were selected to converge with the existing literature. Following literature reviews (Olofsson et al., 2008; Hajcak et al., 2010) and visual inspection of the grand average ERP waveform of the present sample, LPP amplitudes were averaged between 320 and 1500 ms at Pz, PO3/PO4, P3/P4, and CP1/CP2.

### Statistical analyses

A mixed model analysis of variance (ANOVA) was used to estimate the proportion of variability in any given dependent measure that can be attributed to each of the terms in Equation 1. More specifically, we analyzed variability of state asymmetries (F4-F3: range −2.97–5.48, *M* = 0.52, *SD* = 0.99, *n* = 1050; F8-F7: range −5.28–5.04, *M* = −0.19, *SD* = 1.62, *n* = 990), valence ratings (range 1–8.9, *M* = 4.8, *SD* = 2.0, *n* = 1050); arousal ratings (range 1–9, *M* = 5.0, *SD* = 2.0, *n* = 1050) as well as LPP amplitudes (range −13.2–27.5, *M* = 9.2, *SD* = 6.0, *n* = 1050). For each dependent measure the 15 means (5 affective categories ^*^ 3 repetitions) available for each participant were concatenated vertically and subjected to a mixed model ANOVA in the General Linear Model module of Statistical 8.0 software (StatSoft, USA). All ANOVAs included random effects for (a) Participant; (b) Affective category; (c) Participant by category interaction as well as (d) error (corresponding, respectively, to *I*, *S*, *SI*, and ε components of Equation 1). The three repetitions were not factored but instead used as a source of error variance for estimating the interaction term. Two types of results were obtained from each ANOVA—statistical significance of each effect as well as the proportion of dependent measure variance that could be attributed to any given effect (calculated using Analysis of Variance estimation; Searle et al., 2009). Tukey honest significant differences (HSD) test was used for *post-hoc* pair-wise comparisons between affective categories.

## Results

### Subjective ratings and LPP amplitudes

Table 1 presents results of the mixed model ANOVAs of subjective affective ratings and LPP amplitudes. The moderate to high values of the adjusted *R*^2^ statistic suggest that the analysis models provided adequate fit to neural and in particular to the ratings data. Note also that each of the three substantial components of Equation 1—stimulus main effect (*S*), stimulus-independent individual differences (*I*) as well as stimulus-dependent individual differences (*SI*)—made significant contributions to each analyzed measure.

**Table 1:** **Results of mixed model ANOVAs of affective ratings and LPP amplitudes**.

	**Valence**					**Arousal**				**LPP**			
	***df***	***F***	***p***	**η_*p2*_**	**%**	***F***	***p***	**η_*p2*_**	**%**	***F***	***p***	**η_*p2*_**	**%**
Intercept	1	29.1	0.003	0.88		60.3	0.001	0.93		68.8	0.001	0.92	
Affect	4	406.0	0.001	0.85	83.3	127.6	0.001	0.65	46.5	51.8	0.001	0.43	12.9
Participant	69	1.3	0.05	0.25	1	5.7	0.001	0.59	23.9	14.1	0.001	0.78	46.5
Affect * Participant	276	21.7	0.001	0.90	13.7	13.2	0.001	0.84	23.8	1.6	0.001	0.38	6.3
Error	700				2				5.8				34.3

Whole model adjusted R^2^ was 0.98 for valence ratings, 0.94 for arousal ratings, and 0.65 for LPP amplitudes.

η_**p2**_—partial eta squared; %—proportion of variance accounted for.

**Figure 1:**
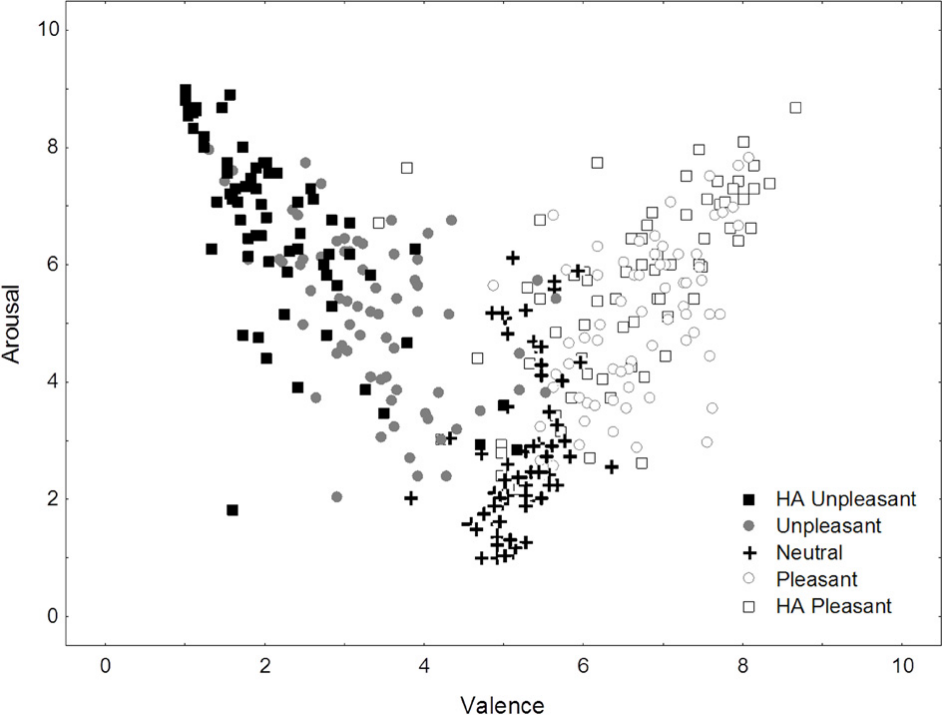
**Individual mean valence and arousal ratings for the affective categories presented in this study**.

The significant affective category main effects in both affective rating dimensions confirmed that subjectively different affective experiences were elicited in this experiment (see Figure 1). *Post-hoc* analyses revealed only a slight deviation from the expected ratings pattern whereby the states elicited by HA pleasant images were (a) equally rather than more positive than responses to low arousal pleasant images as well (b) less rather than as arousing as the ones generated by HA unpleasant stimuli. Specifically, mean valence ratings increased from HA unpleasant (*M* = 2.15) via unpleasant (*M* = 3.42) and neutral (*M* = 5.20) to HA pleasant (*M* = 6.53) and then pleasant categories (*M* = 6.75, all *SE* = 0.10; all pair-wise Tukey HSD test *p* < 0.001). Arousal ratings meanwhile increased from neutral (*M* = 2.75) via a level shared by pleasant (*M* = 5.05) and unpleasant images (*M* = 5.16, *p* = 0.15) to HA pleasant (*M* = 5.53) and HA unpleasant stimuli (*M* = 6.65, all *SE* = 0.13; all remaining pair-wise comparisons *p* < 0.001). In summary, participant self-reports indicated successful elicitation of several distinct types of core affect/biphasic motivation.

**Figure 2:**
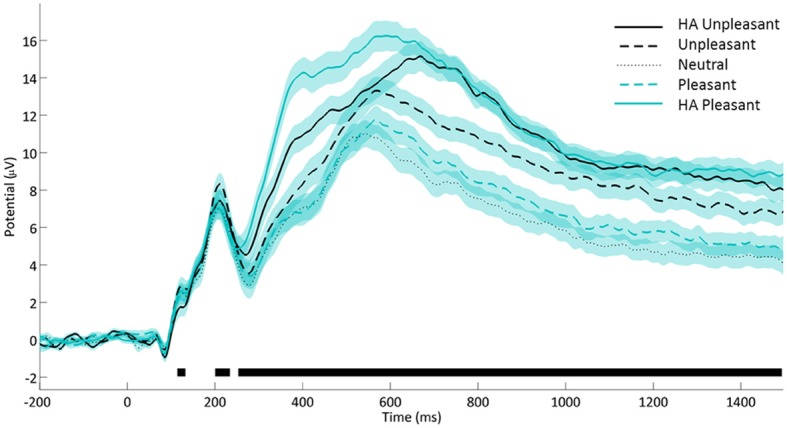
**Affective modulation of the late positive potential.** Notes. Average voltage from CP1/CP2, P3/P4, PO3/PO4, and Pz. Shaded areas denote standard errors. Black lines denote time points with significant affective category main effect (ANOVA, false discovery rate corrected *p* < 0.05).

Analyses of LPP amplitudes also confirmed an expected pattern (see Figure 2) whereby neutral (*M* = 6.63) and pleasant (*M* = 7.38) stimuli elicited similar amplitudes (*p* = 0.19) below the responses to unpleasant images (*M* = 9.28) which in turn preceded the largest amplitudes induced by HA unpleasant (*M* = 10.95) together with HA pleasant stimuli (*M* = 11.77, *p* = 0.13; all *SE* = 0.31, all remaining pair-wise comparisons *p* < 0.001). Note that the LPP amplitudes and subjective arousal ratings diverged in two respects. The LPP response to HA pleasant stimuli exceeded the level generated by HA unpleasant images while the reverse was true for arousal ratings. Similarly, pleasant images shared a response level with neutral stimuli within the LPP amplitudes while they were closer to the unpleasant category in terms of arousal ratings. This pattern suggests that LPP amplitudes and subjective arousal ratings are not redundant measures, justifying their parallel analysis.

### State asymmetries

**Figure 3:**
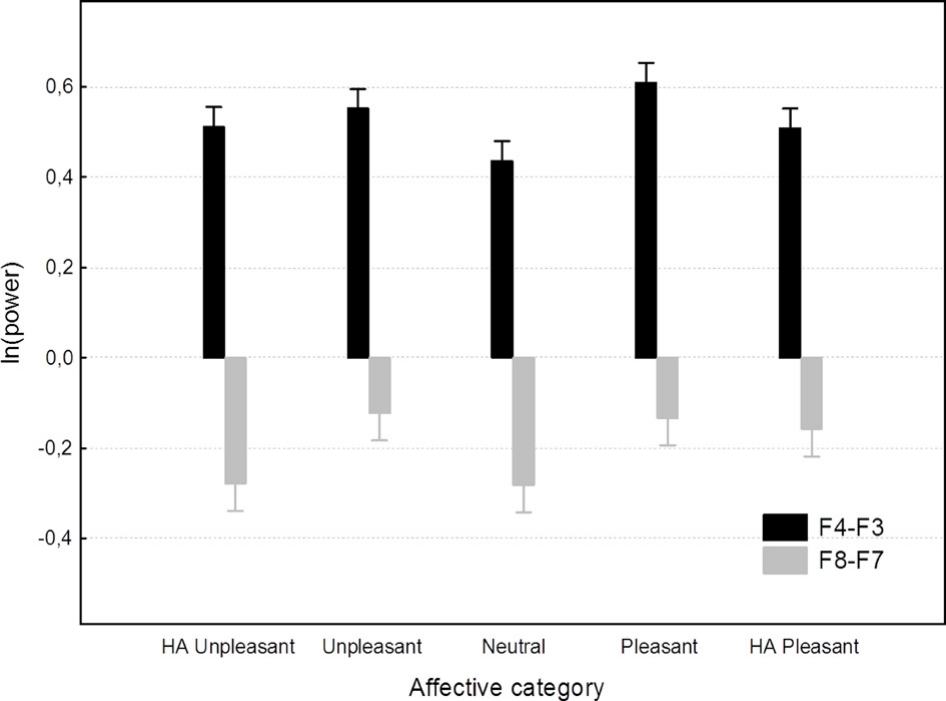
**Affective category main effect on F4-F3 and F8-F7 asymmetries.** Spreads denote standard errors.

Results of the mixed model ANOVAs of F4-F3 as well as F8-F7 asymmetries are shown in Table 2 and illustrated on Figure 3. Unlike the subjective ratings as well as LPP amplitudes, state asymmetries recorded in this study were completely insensitive to affective images—the stimulus main effect remained insignificant and accounted for only a small fraction of overall variance. *Post-hoc* analyses of the affective main effect meanwhile revealed that none of the pair-wise contrasts reached significance (Tukey HSD *p* = 0.06 for positive vs. HA pleasant F4-F3 asymmetry; *p* > 0.30 for all other comparisons). Trend level differences between asymmetries elicited by different picture categories also failed to align with the prediction that asymmetry should correlate with the valence of the stimuli (see Figure 3). Instead, all images elicited quite uniform asymmetries which were positive for the F4-F3 and negative for the F8-F7 location. These findings clearly replicate previously reported difficulties to find expected stimulus main effects in experiments employing affective images.

**Table 2:** **Results of mixed model ANOVAs of F4-F3 and F8-F7 state asymmetries**.

	**F4-F3 asymmetry**					**F8-F7 asymmetry**				
	***df***	***F***	***p***	**η_*p2*_**	**%**	***df***	***F***	***p***	**η_*p2*_**	**%**
Intercept	1	31.4	0.001	*0.32*		1	1.6		*0.02*	
Affect	4	2.1		*0.03*	0.2	4	1.7		*0.03*	0.1
Participant	69	21.7	0.001	*0.84*	54.8	65	30.2	0.001	*0.88*	67.5
Affect * Participant	276	0.9		*0.26*	0.0	260	1.1		*0.30*	1.1
Error	700				45.0	660				31.2

Whole model adjusted R^2^ was 0.54 for F4-F3 asymmetry and 0.69 for F8-F7 asymmetry.

η_p2_—partial eta squared; %—proportion of variance accounted for.

**Figure 4:**
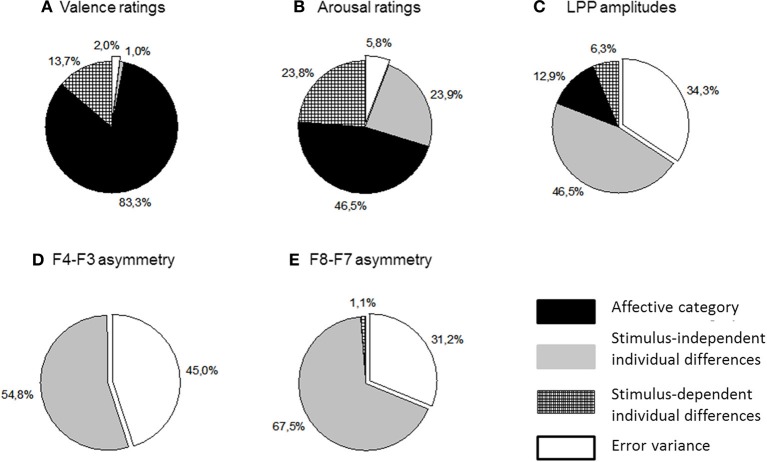
**Proportions of variance accounted for by the affective category and two types of individual differences (see Equation 1) in subjective valence (panel A) and arousal ratings (panel B); LPP amplitudes (panel C) and two asymmetry estimates (panels D,E)**.

Table 2 lists and Figure 4 plots the proportions of variance in all analyzed dependent measures accounted for by each component of Equation 1. These analyses revealed no significant stimulus-dependent individual differences at either asymmetry location suggesting that the patterns of asymmetries elicited by picture categories did not differ significantly across participants. This finding implies that individual differences in asymmetry responsiveness cannot explain the lack of the affective main effect. By contrast, stimulus-dependent individual differences made sizeable contributions to arousal ratings as well as LPP dynamics. Figure 4 also illustrates that the affective category was the largest source of variability for both types of ratings, valence in particular. It also contributed significantly to LPP variance. All EEG measures meanwhile were dominated by stimulus-independent individual differences. However, unlike the LPP amplitudes which showed significant contributions from all sources, asymmetries recorded in this study were significantly influences only by stimulus-independent individual differences.

## Discussion

The problem addressed in this article stems from juxtaposing three widely accepted ideas—that affective perception activates core affect (Bradley and Lang, 2007); that core affect and biphasic motivation are unlikely to occur in isolation (Lang, 2010); and that anterior EEG asymmetry reflects biphasic motivation (Harmon-Jones et al., 2010). By logical inference, affective perception should therefore generate shifts in state asymmetry. However, complementing earlier studies (Elgavish et al., 2003; Harmon-Jones et al., 2006; Gable and Harmon-Jones, 2008; Huster et al., 2009; Gable and Poole, 2012) the present results raise the troubling possibility that it sometimes does not. To limit the number of possible explanations for that conundrum, we tested if the present null findings could be attributed to insufficient motivational intensity and/or stimulus-dependent individual differences. Neither hypothesis was supported by our analyses. Subjective affective ratings as well as LPP amplitudes suggested that the images successfully induced variable forms of core affect. Mixed model ANOVAs meanwhile demonstrated that asymmetry was insensitive to stimuli on sample as well as participant average levels. In this section we will discuss these observations in more detail before suggesting hypothesis for future attempts to resolve the affective images conundrum in asymmetry literature.

### Sufficient motivational intensity

It has been suggested that the states induced by viewing affective images lack the motivational intensity required for asymmetry effects to occur (Harmon-Jones et al., 2006; Gable and Harmon-Jones, 2008). We conducted a simple test of this idea. If the absence of anterior asymmetry effects in the present data reflected the lack of motivational relevance of the stimuli, then the stimuli should also not modulate LPP amplitudes nor differ significantly in terms of subjective ratings. Quite contrary to this hypothesis, a clear dissociation emerged between asymmetry on one hand, and self-report as well as neural measures of motivational intensity on the other. Insofar as valence ratings reflect the direction, and arousal ratings the intensity of affective-motivational states (Bradley and Lang, 2007), the self-report differences between picture categories gave the first indication of successful core affect/biphasic motivation manipulation. Note that as subjective ratings may reflect nothing more than correct cognitive categorization of stimuli, they alone offer only limited proof for the occurrence of affective processing. However, when considered together with the present LPP findings, it is much more likely that the stimuli used in this study were indeed felt to be motivationally significant (Schupp et al., 2006; Sabatinelli et al., 2007, 2013; Hajcak et al., 2010). In line with previous findings, we observed larger LPP amplitudes in response to most affective stimuli compared to the nonaffective neutral category. Furthermore, two distinct levels of LPP amplitudes emerged corresponding roughly to the intended distinction between low and high arousing categories. Thus, even if the less arousing unpleasant and in particular pleasant images indeed generated only weak affective states with insufficient motivational intensity, the same is unlikely to be true for the highly arousing pleasant and unpleasant pictures. In summary, the significant and expected modulations of subjective as well as brain-based measures of motivational intensity obtained concurrently with anterior asymmetry make it unlikely that core affect, and by extension, some level of approach-avoidance motivation was unsuccessfully induced in the present study.

Even while our findings suggest that participants perceived the stimuli to be motivationally significant, we have much less information about action-related components of biphasic motivation induced in this experiment. These components are potentially relevant, however, as motivational states are known to be dependent on the availability of congruent actions. For instance, the brain response to smoking cues is diminished, although not abolished, when participants know they cannot smoke for several hours (Wilson et al., 2005). In the present study, participants were also unable to overtly avoid or approach the presented stimuli, which might have diminished their motivation to do so. Such characteristics of the available action repertoire can also influence asymmetry. For instance, asymmetry responses to anger stimuli were reduced in a supine body position compared to an upright, more approach-conducive posture (Harmon-Jones and Peterson, 2009). On the other hand however, several studies suggest that asymmetry can remain independent of actions. In a rewarded delayed reaction paradigm, expected asymmetries were recorded irrespective of the motivational congruency of required responses (e.g., press vs. release a button to approach; Sobotka et al., 1992) as well as when no overt responses were required from participants (Miller and Tomarken, 2001). These findings suggest that asymmetry effects should have occurred in the present experiment despite the lack of motivationally congruent action requirements. In summary, further research is needed to determine how and when action repertoire influences anterior asymmetry. For now, it is probably safe to conclude that even while the availability of actions can change motivational states, it is unlikely to completely obliterate them and, by extension asymmetry effects. It thus remains questionable if the current null finding can be explained solely by the lack of explicit action requirements.

### Stimulus-dependent individual differences

Another feasible explanation for the affective images conundrum is the idea that a sample-level null effect can conceal expected asymmetries exhibited by single participants (Harmon-Jones et al., 2006, 2010, 2011; Gable and Harmon-Jones, 2008, 2013). However, the mixed model analyses conducted in this study demonstrated that stimulus-dependent individual differences did not have a detectable effect on asymmetry. In other words, participants did not systematically differ in their asymmetry responses to stimulus categories. This pattern is of particular significance given that the analysis strategy we adopted did not require *a priori* identification of the states or traits that would be most relevant for asymmetry. We thus maximized the likelihood of discovering stimulus-dependent individual differences in asymmetry responsiveness. Nevertheless, we found asymmetry to be determined by stimulus-independent interindividual variance indicating that large differences between participants were measured irrespective of the type of stimulus being presented.

Note that we did not explicitly test the possibility that individuals responded differently to specific pictures rather than whole stimulus categories. Indeed, this version of the individual differences hypothesis cannot be ruled out for the semantically heterogeneous low arousing pleasant and unpleasant categories. However, the high arousing unpleasant and pleasant categories were semantically relatively consistent containing, respectively, only depictions of mutilated human bodies and heterosexual nude couples. Individually specific responses to these particular types of pictures (e.g., males being more attracted by erotica than females) would thus have resulted in significant stimulus-dependent individual differences in mixed model analyses of asymmetry. The fact that they did not, combined with individual differences observed for both affective ratings and LPP amplitudes, suggests that the individually different affective responsiveness exhibited by the present sample remained invisible in anterior asymmetry. Idiosyncratic responses to stimuli thus also remain an unlikely explanation for the lack of affective asymmetry effects in the present study.

### Alternative explanations

The preceding discussion suggests that existing explanations for the affective images conundrum in asymmetry literature have difficulties in accounting for present findings. Meanwhile, the large sample and availability of around 50 s of EEG for each affective category reduce the risk that the current results reflect limited statistical power or low signal to noise ratio. Consequently, alternative explanation may be required for the absence of expected asymmetry effects in response to affective picture stimuli. The fact that asymmetry variance was dominated by stimulus-independent individual differences raises two hypotheses in this regard. As the present data were measured in a single experimental session, this variance component may reflect any combination of two types of individual differences. On one hand, it may capture stimulus- as well as situation-independent, essentially trait-like asymmetry differences between participants. On the other, it could also reflect stimulus-independent yet situation-dependent interindividual state-like variance. Both conceptualizations may open up new ways of thinking about the current and previous null findings as well as anterior asymmetry in general.

If true trait-like variance were involved, then the null findings could simply stem from the fact that anterior asymmetry is dominated by stable individual differences rather than experimentally induced shifts (see also Levy et al., 1983; Tomarken et al., 1992; Towers and Allen, 2009). Or, in a more nuanced version of this possibility, changes in state asymmetry may occur but remain inherently unreliable due to an order of magnitude larger trait variance noise overshadowing the state signal. Alternatively, stimulus-independent but situation-dependent variance may also have determined the asymmetry variability in this experiment. In this case asymmetry theorists should consider individual differences elicited by the experimental situation as a whole, rather than the stimuli as potential generators of asymmetry effects. For instance, encounters with highly arousing pleasant and, in particular unpleasant images were unpredictable in the present experiment. This might have activated spontaneous emotion regulation or anxious apprehension in varying degrees in different participants. Both mechanisms could conceivably alter brain activity throughout the whole experiment generating situation-dependent but stimulus-independent individual differences. Emotion regulation is known to rely on prefrontal brain regions (Phillips et al., 2008) and has sometimes been implicated in asymmetry research (Tomarken and Keener, 1998; Jackson et al., 2003; Dennis and Solomon, 2010; Kim et al., 2012; Parvaz et al., 2012). Anxious apprehension meanwhile has also been linked to specific patterns of anterior asymmetry as well as prefrontal brain activity (Engels et al., 2007; Crost et al., 2008; Stewart et al., 2008). Future studies, preferably employing several measurement occasions, are required to isolate different contribution to stimulus-independent individual differences.

## Summary and conclusions

In this paper, the absence of expected asymmetry responses to affective images was replicated using lengthy data from a large sample. Concurrently measured subjective ratings and LPP amplitudes indicated that this did not result from a lack of motivational relevance of the stimuli. Mixed model ANOVAs meanwhile revealed that individual differences in responsiveness to stimulus categories also struggle to explain this null finding. Taken together with previous reports, these results suggest that alternative explanations are required for the affective images conundrum in asymmetry literature. Two hypotheses for this purpose were derived from the fact that the observed asymmetries were dominated by stimulus-independent individual differences. First, trait asymmetry may constitute a large source of noise rendering the study of state effects inherently difficult. Alternatively, sustained responses to the experimental settings such as spontaneous emotion regulation or anxious apprehension may be involved in generating anterior asymmetry. While studies confirming or rejecting these hypotheses remain to be conducted, it seems prudent to maintain the possibility that under some circumstances true shifts in approach-avoidance motivation may remain invisible in anterior asymmetry.

## Author contributions

Andero Uusberg, Helen Uibo, Helena Sarapuu, Kairi Kreegipuu, and Jüri Allik conceived and designed the experiment. Andero Uusberg, Helen Uibo, Helena Sarapuu and Riti Tiimus conducted the experiments and developed the aims of the paper. Andero Uusberg analyzed the data and wrote of the first draft. Andero Uusberg, Helen Uibo, Kairi Kreegipuu, and Jüri Allik contributed to the final draft.

### Conflict of interest statement

The authors declare that the research was conducted in the absence of any commercial or financial relationships that could be construed as a potential conflict of interest.
